# Computed tomography and magnetic resonance imaging findings in infants with microcephaly potentially related to congenital Zika virus infection

**DOI:** 10.1590/0100-3984.2016.0135

**Published:** 2018

**Authors:** Aníbal Araujo Alves Peixoto Filho, Simone Baltar de Freitas, Márcio Morikoshi Ciosaki, Lourenço Nogueira e Oliveira, Onildo Tavares dos Santos Júnior

**Affiliations:** 1 MD, Specialist in Radiology and Diagnostic Imaging for the Rede Sarah de Hospitais de Reabilitação, Salvador, BA, Brazil.

**Keywords:** Microcephaly, Zika virus infection, Tomography, X-ray computed, Magnetic resonance imaging, Microcefalia, Infecção pelo vírus Zika, Tomografia computadorizada, Ressonância magnética

## Abstract

The recent association between the increase in the number of neonates with microcephaly in northeastern Brazil and the outbreak of infection with the Zika virus, which has been occurring in the Americas, has been declared a public health emergency of international concern. The evidence that implicates the virus as the cause of this public health emergency has been demonstrated ever more consistently. This pictorial essay illustrates the imaging characteristics seen on computed tomography and magnetic resonance imaging scans of infants admitted to a rehabilitation hospital with a diagnosis of microcephaly and a maternal history of rash during pregnancy.

## Abstract (pt)

A recente associação entre aumento do número de nascidos vivos com microcefalia no nordeste brasileiro com o surto pelo vírus Zika, que vem ocorrendo nas Américas, está sendo considerada uma emergência em saúde pública de interesse internacional. As evidências que apontam o vírus como causador deste agravo em saúde pública vêm sendo definidas de forma cada vez mais consistente. Este ensaio demonstra características de imagem de tomografia computadorizada e ressonância magnética em bebês admitidos em um hospital de reabilitação, com diagnóstico de microcefalia e antecedentes maternos de doença exantemática durante a gestação.

## INTRODUCTION

States in several parts of the northeastern region of Brazil have recorded an unusual increase in the number of infants born with microcephaly, an urgent public health problem that affects the quality of life of children and their families. The situation has recently been declared a public health emergency of international concern^(1)^.

Zika virus infection in humans was first identified in Uganda and Tanzania in 1952, subsequent outbreaks being reported in other parts of Africa, as well as in the Americas, Asia, and the Pacific^(2)^. However, only recently, in large outbreaks in French Polynesia in 2013 and in Brazil in 2015, neurological and autoimmune complications have been reported, as has the current association with microcephaly^(3,4)^.

The elucidation of the etiological factor responsible for this most recent outbreak involves, among other factors, the recognition of the relationship between the presence of the virus and the occurrence of microcephaly; verification of the virus crossing the placental barrier; and identification of the Zika virus in stillborn and newborn infants with microcephaly or other malformations of the central nervous system^(3)^. The virus has been identified in the amniotic fluid of pregnant women following the ultrasound diagnosis of microcephaly^(5)^, in the brain tissue of a fetus aborted at 32 weeks of gestation in Slovenia^(4)^, and in a neonate that evolved to death in the northeastern Brazilian state Ceará^(3)^.

According to the World Health Organization^(6)^, imaging examinations are necessary for the detection of structural abnormalities in the brains of children born with microcephaly.

## EMBRYOLOGY

In brief, the complex embryological process of brain development involves neurulation, followed by neuronal proliferation, neuronal migration, organization (formation of the opercula, sulci, and gyri), and, finally, myelination. That process begins in the third week of gestation and continues until after birth. The process can be impeded by events of different natures, which, depending on the severity of the insult, can cause microcephaly. Microcephaly can be primary (genetic) or secondary, the most common causes of secondary microcephaly being vascular disorders, maternal diabetes, trauma, and infections^(7)^.

## CLINICAL ASPECTS

The images presented in this study were obtained from the examinations of nine infants admitted to a rehabilitation hospital with microcephaly apparently related to congenital Zika virus infection, according to the criteria established by local authorities^(3)^.

The infants studied were between 1 and 7 months of age at the time of the examination. All of their mothers had reported having had a cutaneous rash between the second and fourth months of gestation. The serology performed during pregnancy did not identify another infectious factor that could be the cause of the microcephaly. The infants underwent computed tomography (CT) and magnetic resonance imaging (MRI) without sedation, with guidelines related to sleep deprivation.

## IMAGING FINDINGS

Various congenital infections, although not pathognomonic, share some imaging characteristics, chief among which are calcifications. Other findings, such as ventriculomegaly, posterior fossa alterations, and malformations of cortical development, have been reported^(3)^. Below, we describe the main imaging findings among our patients with microcephaly.

### Parenchymal calcifications

**Figure 1:**
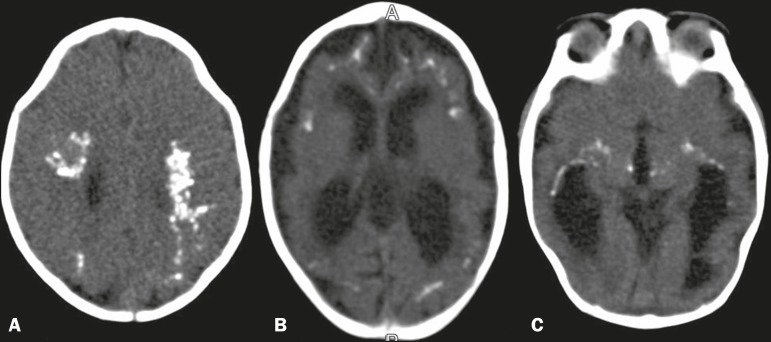
CT scan showing parenchymal calcifications in several locations in different patients. A: In the deep white matter and in cortical-subcortical regions, at the level of the corona radiata, assuming a confluent aspect. B: In cortical-subcortical regions, at the level of the lateral ventricles. C: Calcifications in the thalamus and in the capsulonuclear regions. Note the narrowing of the frontal bones, in A, and the small left frontal subcortical cyst, in B.

**Figure 2:**
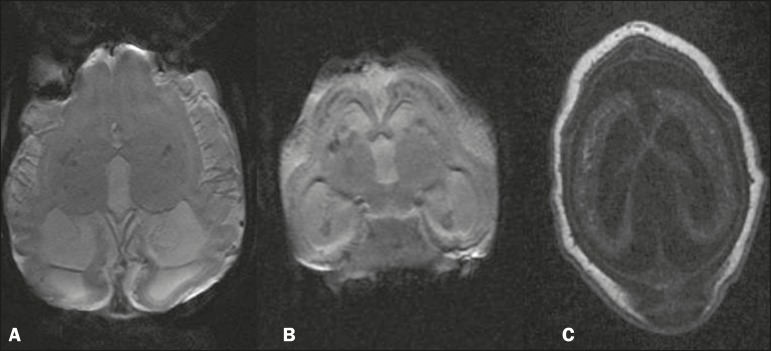
MRI. A,B: Volumetric gradient-echo sequences showing foci of signal hypointensity in the cortical-subcortical regions, thalamic, and capsulonuclear regions. C: Volumetric fast spoiled gradient-echo T1-weighted sequence, of the same patient depicted in B, showing foci of signal hyperintensity, confluent in the cortical-subcortical regions, corresponding to the foci of signal hypointensity seen in the gradient-recalled echo sequence.

In all of the patients included, we observed parenchymal calcifications, varying in number and location, that were characterized by hyperattenuating foci on CT scans (Figure 1) and by areas of low signal intensity in gradient-echo MRI sequences (Figure 2). Some of the calcifications also showed high signal intensity in fast spin-echo and fast spoiled gradient echo T1-weighted sequences.

Calcifications were often seen in cortical-subcortical regions, either in a linear arrangement or confluent (Figure 1A). Several calcifications were also identified in the deep white matter, periventricular white matter, and deep gray matter (Figure 1C).

### Alterations in the morphology of the parenchyma and gyri

**Figure 3:**
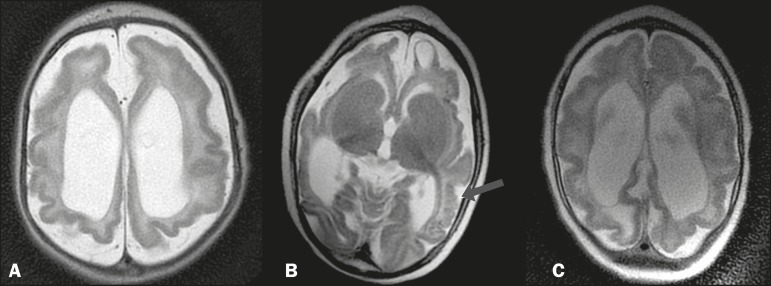
MRI, T2-weighted sequences in different patients, showing a pattern of gyral simplification, accompanied by ventriculomegaly and enlargement of the subarachnoid spaces. In B, a subcortical cyst can be seen in the left frontal region, as can polymicrogyria, which is best visualized in the left temporoparietal region (arrow).

**Figure 4:**
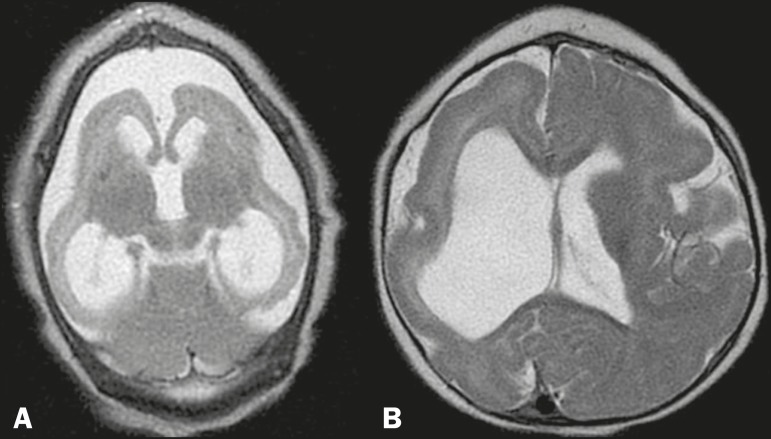
MRI scan showing patterns of altered gyral morphology on the lissencephaly spectrum, characterized by smoother contours of the cortical surface and a certain thickening, with a more generalized presentation in A and an asymmetric presentation in B, with more pronounced pachygyria in the right frontal region.

We identified alterations in the gyral pattern, a simplified pattern, characterized by few gyri and shallow sulci, predominating throughout the parenchyma (Figure 3). We also identified alterations on the lissencephaly spectrum (Figure 4), with cortical thickening, greater scarcity (or even the absence) of gyri, and a nearly smooth parenchymal surface, seen in a more diffuse or localized form. In other infants, we observed areas of polymicrogyria accompanied by gyral simplification (Figure 3B). Several patients presented a marked reduction in parenchymal volume, ventriculomegaly being quite common and, in some cases, accompanied by enlargement of the subarachnoid spaces.

### Alterations in the corpus callosum

**Figure 5:**
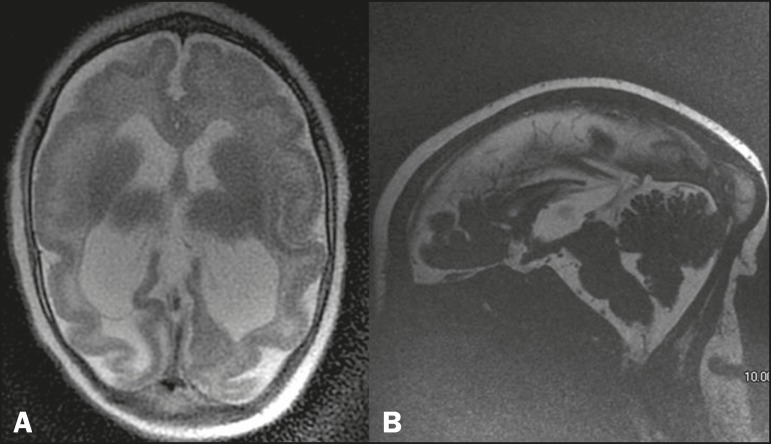
MRI: axial T2-weighted sequence (A) showing hypogenesis of the corpus callosum; and sagittal three-dimensional fast imaging employing steady-state acquisition sequence (B), showing more clearly the genu and body of the corpus callosum, both of which are hypoplastic.

**Figure 6:**
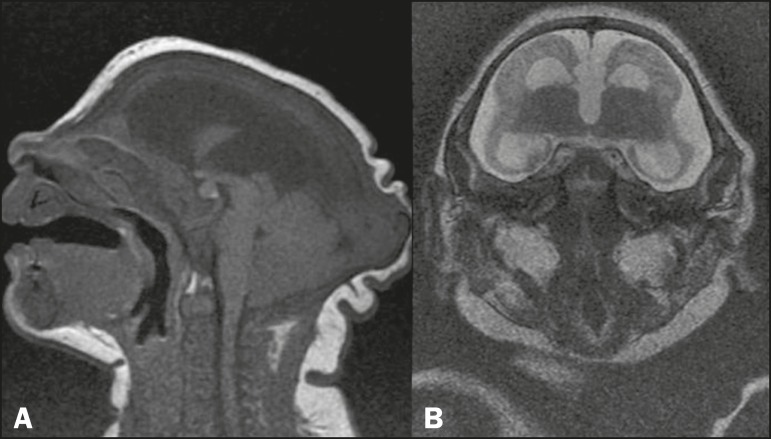
MRI: sagittal T1-weighted sequence (A) and coronal T2-weighted sequence (B), both showing agenesis of the corpus callosum. In A, note the craniofacial disproportion, occipital bone protuberance ("saddling"), skin folds, and caudal projection of the cerebellar tonsils. In B, note the high positioning of the third ventricle and the absence of the corpus callosum, as well as enlargement of the subarachnoid space, ventriculomegaly, and considerable reduction in the size of the telencephalon.

In all of the examinations included in this study, we observed changes in the corpus callosum, characterized by hypogenesis (Figure 5) or agenesis (Figure 6), with or without significant hypoplasia. Consequently, in some cases-those in which the agenesis or hypogenesis was more pronounced-there was a predominance of colpocephaly of the lateral ventricles (Figure 5A).

### Other alterations

**Figure 7:**
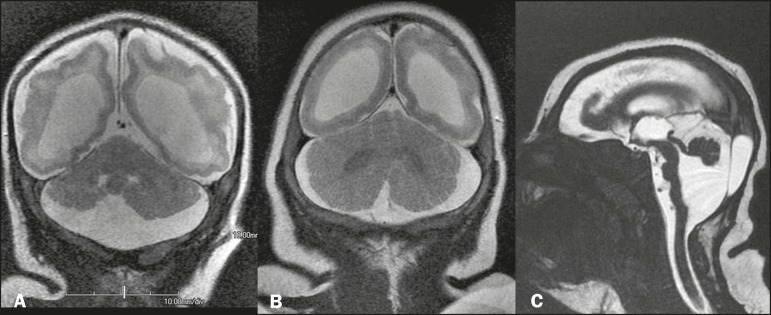
MRI. A,B: Coronal T2-weighted sequence showing discrete hypoplasia of the right cerebellar hemisphere (in A) and relative prominence of the cerebellum in relation to the telencephalon (in B), as well as (in both) ventriculomegaly and a significant reduction in the volume of the white matter. C: Hypoplasia and rotation of the cerebellar vermis, accompanied by a prominent cerebrospinal fluid collection in the posterior fossa, as well as changes on the Dandy-Walker continuum. Note that there is also hypoplasia of the brainstem, notably of the pons.

We identified changes in the posterior fossa (Figure 7), including hypoplasia of the right cerebellar hemisphere and some changes on the Dandy-Walker continuum. In one of the infants, we observed a subcortical cyst (Figure 3B). In the cranial vault, we identified occipital protuberance (Figures 5B and 6A) and narrowing of the frontal bones (Figure 1A).

## CONCLUSION

Due to the significant increase in the number of infants born with microcephaly, neuroimaging examinations have become an indispensable tool for investigating the morphology of the brain parenchyma of such infants. In this essay, we have illustrated the main imaging findings, in the initial examinations carried out at our institution, in infants with microcephaly that was apparently related to congenital infection with the Zika virus.
